# Higher incidence of death in multi-vessel coronary artery disease patients associated with polymorphisms in chromosome 9p21

**DOI:** 10.1186/1471-2261-12-61

**Published:** 2012-08-02

**Authors:** Luciana Gioli-Pereira, Paulo Caleb Junior Lima Santos, Noely Evangelista Ferreira, Whady Armindo Hueb, Jose Eduardo Krieger, Alexandre Costa Pereira

**Affiliations:** 1 Laboratory of Genetics and Molecular Cardiology, Heart Institute (InCor), Sao Paulo University Medical School, Av. Dr. Enéas de Carvalho Aguiar, 44 Cerqueira César, Sao Paulo, SP, Brazil

**Keywords:** Coronary artery disease, Polymorphism, Genetics, Chromosome 9p21

## Abstract

**Background:**

We investigated whether 9p21 polymorphisms are associated with cardiovascular events in a group of 611 patients enrolled in the Medical, Angioplasty or Surgery Study II (MASS II), a randomized trial comparing treatments for patients with coronary artery disease (CAD) and preserved left ventricular function.

**Methods:**

The participants of the MASS II were genotyped for 9p21 polymorphisms (rs10757274, rs2383206, rs10757278 and rs1333049). Survival curves were calculated with the Kaplan–Meier method and compared with the log-rank statistic. We assessed the relationship between baseline variables and the composite end-point of death, death from cardiac causes and myocardial infarction using a Cox proportional hazards survival model.

**Results:**

We observed significant differences between patients within each polymorphism genotype group for baseline characteristics. The frequency of diabetes was lower in patients carrying GG genotype for rs10757274, rs2383206 and rs10757278 (29.4%, 32.8%, 32.0%) compared to patients carrying AA or AG genotypes (49.1% and 39.2%, p = 0.01; 52.4% and 40.1%, p = 0.01; 47.8% and 37.9%, p = 0.04; respectively).

Significant differences in genotype frequencies between double and triple vessel disease patients were observed for the rs10757274, rs10757278 and rs1333049. Finally, there was a higher incidence of overall mortality in patients with the GG genotype for rs2383206 compared to patients with AA and AG genotypes (19.5%, 11.9%, 11.0%, respectively; p = 0.04). Moreover, the rs2383206 was still significantly associated with a 1.75-fold increased risk of overall mortality (p = 0.02) even after adjustment of a Cox multivariate model for age, previous myocardial infarction, diabetes, smoking and type of coronary anatomy.

**Conclusions:**

Our data are in accordance to previous evidence that chromosome 9p21 genetic variation may constitute a genetic modulator in the cardiovascular system in different scenarios. In patients with established CAD, we observed an association between the rs2383206 and higher incidence of overall mortality and death from cardiac causes in patients with multi-vessel CAD.

## Background

Coronary heart disease (CHD), including acute myocardial infarction, are leading causes of morbidity and mortality worldwide [1]. Lifestyle and environmental factors play an important role in their development, but genetic inheritance appears to be strongly involved [2]. Recently, genome-wide association studies have revealed single nucleotide polymorphisms (SNP) on chromosome 9p21 that confer susceptibility to coronary artery disease (CAD) and myocardial infarction (MI) in some populations such as Caucasians from Northern Europe [3], North American [4], Italian [5] and Belgian [6]. Among the Han Chinese population, Lin et al. observed that the chromosome 9p21 had a significant association with carotid atherosclerosis in a gender-specific manner [7]. Moreover, meta-analysis of the relationship between chromosome 9p21.3 polymorphisms and CAD has provided stronger evidences for the association [8,9].

9p21.3 is a chromosomal region relatively replete of open reading frames (ORF) and the closest genes are a cluster consisting of CDKN2A-ARF-CDKN2B (cyclin-dependent kinase N2A and N2B), MTAP, and ANRIL. The linkage disequilibrium (LD) structure of this genomic locus varies depending on the studied population, and different LD blocks are described between associated SNP and ORF in the region. This locus has been associated with tumor suppression, cell proliferation, senescence, apoptosis [10,11], all features implicated in atherogenesis and now with CAD [12]. The mechanisms involved remain unclear but studies in mice have provide direct evidence that the CAD risk interval has a pivotal role in regulation of cardiac Cdkn2a/b expression, and suggest that this region affects CAD progression by altering the dynamics of vascular cell proliferation [13].

At the same time, genetic variants around the same genes were associated with an increased risk of type 2 diabetes mellitus [14-16] atherothrombosis [17] and ischemic stroke [18]. All these data suggest that there may be common pathogenic mechanisms involved in these apparently disparate diseases. On the other hand, Plant et al. showed that there was no association between rs10757278 and stroke status based on the presence or absence of angiographically demonstrated CAD in non-stroke controls. They supposed an association of this genomic region with ischemic stroke independent of its effect on CAD, suggesting an additional stroke-specific pathophysiological relationship [19].

In this report, we analyzed whether the rs10757274, rs2383206, rs10757278 and rs1333049 polymorphisms are associated with cardiovascular events in a group of 611 patients enrolled in the MASS II [20,21], a study that compared therapeutic strategies for individuals with multi-vessel CAD established. This will explore the association of 9p21 genetic variants in individuals with already established and severe CAD.

## Methods

### Study population

Studied patients were selected from the prospective, randomized and controlled Medicine, Angioplasty, or Surgery Study II (MASS II), which was designed to compare medical treatment, angioplasty or stent (percutaneous coronary intervention, PCI), and myocardial revascularization (coronary artery bypass grafting, CABG) in patients with multi-vessel stable CAD with preserved left ventricular function [20,21].

Methodological details were described by Hueb et al. Briefly, patients with angiographically documented proximal multivessel coronary stenosis of more than 70% by visual assessment and documented ischemia were considered for inclusion. Ischemia was documented by either stress testing or the typical stable angina assessment of the Canadian Cardiovascular Society (CCS) (class II or III). Patients were enrolled and randomized if there was agreement on the part of the surgeon and interventionist that revascularization could be attained by either strategy. Exclusion criteria included unstable angina or acute MI requiring emergency revascularization, ventricular aneurysm requiring surgical repair, left ventricular ejection fraction of <40%, a history of PCI or CABG, and single-vessel disease. Patients were also excluded: if they had a history of congenital heart disease, valvular heart disease, or cardiomyopathy; if they were unable to understand or cooperate with the protocol requirements or to return for follow-up; or if they had left main coronary artery stenosis of 50% or more, or suspected or known pregnancy or another coexisting condition that was a contraindication to CABG or PCI. In this trial, all patients were placed on an optimal medical regiment consisting of a stepped-care approach using nitrates, aspirin, beta-blockers, calcium channel blockers, angiotensin-converting enzyme inhibitors, or a combination of these drugs, unless contraindicated. Hydroxymethylglutaryl-coenzyme A reductase inhibitors were also prescribed, along with a low-fat diet on an individual basis. Patients were then randomized to continue with aggressive medical therapy (MT) alone or to undergo PCI or CABG concurrently with MT [20,21].

Two thousand seventy-six candidates who had indications for myocardial revascularization were evaluated from May 1995 to May 2000. Of these, 611 patients were eligible and met all entry criteria to be randomly assigned to 1 of the 3 therapeutic groups: medical treatment (n = 203), PCI (n = 205), and CABG (n = 203). The primary end-point of this study was to compare the frequencies of major cardiac events, such as acute MI, overall mortality or refractory angina requiring revascularization procedures.

All subjects gave informed consent, and the Ethics Committee of the Heart Institute of the University of Sao Paulo - Brazil approved the study.

### Data collection

The beginning of treatment was considered to be the randomized date and patients were studied by the intention to treat principle. The follow-up time for all patients in this study was 5 years with clinical visits conducted in a 6-month interval. The collected demographic and laboratory data included age, gender, angiographic findings and traditional risk factors, such as history of previous coronary events, hypertension, diabetes, BMI (body mass index), severity of angina, smoking status, and cholesterol and triglycerides profile. Blood samples were obtained from each participant at randomization.

Adverse and other clinical events were tracked from randomization. Patients underwent a symptom-limited treadmill exercise test at baseline and every year until the end of the study, unless contraindicated. Symptoms of angina were graded according to their severity and were considered refractory only when patients had been treated with full anti-ischemic therapies. Myocardial infarction was defined as the presence of significant new Q waves in at least 2 ECG leads or symptoms compatible to MI associated with creatinine kinase-MB elevations. Cardiac mortality was defined when patient died of MI.

### Genotyping

Genomic DNA was extracted from peripheral blood leukocytes by means of a standard salting-out procedure. Genotyping assays were run as submicroliter PCR-based assay on array tape that is a continuous plastic tape used in conjunction with a flexible configuration of dispensing, pipetting, sealing and detection modules manufactured by Douglas Global Array. This method is especially suitable for processes based on PCR and a modified allele-specific PCR assay as described by Myakishev et al. [22] was used. We obtained an average genotyping success rate of more than 95% and an average genotyping accuracy of more than 98% by re-genotyping 32 samples.

### Statistical analysis

Data are presented as mean ± SD for continuous variable and as frequencies for categorical variables. Differences in baseline characteristics among groups were analyzed using ANOVA for continuous variables and chi-square test for categorical variables. Survival curves were calculated with the Kaplan–Meier method and differences between the curves were evaluated with the log-rank statistic. We assessed the relationship between baseline variables and composite end-point events using a Cox proportional hazards survival model adjusted for age, previous MI, diabetes, smoking and type of coronary anatomy. Hazard ratios (relative risks) with 95% confidence intervals (CIs) demonstrate the risk for combined events. Biochemical data and BMI were adjusted for age and gender.

The Hardy-Weinberg equilibrium (HWE) analyses were conducted with Haploview 4.0 for Windows and genotypic distributions for the rs10757274, rs2383206, rs10757278 and rs1333049 polymorphisms were in accordance with the HWE (p = 0.32, p = 0.82, p = 0.64, p = 0.99, respectively). Multiple testing correction was not performed and our sample number only provide 80% power to detect an association with overall mortality with an effect size of 3.8 for the rs10757274 G variant allele, 3.4 for the rs2383206 G variant allele, 3.9 for the rs10757278 G variant allele, and 3.9 for the rs1333049 G variant allele. A value of p ≤ 0.05 was considered significant for comparisons and statistical analyses were performed with SPSS 13.0 for Windows.

## Results

### Baseline clinical characteristics according to genotypes

Of the 611 patients (mean age 60 ± 9), 92 (15.1%) were female and 519 (84.9%) male. Patients in all three therapeutic groups were similar with respect to general characteristics, severity of angina, use of medication, history of MI, diabetes mellitus, or hypertension. The characteristics of 611 patients assigned to one of three groups for multivessel disease are available in previous study [20,21].

The variant allele frequencies were 51%, 59%, 51%, and 48% for the rs10757274 (N = 498), rs2383206 (N = 495), rs10757278 (N = 496), and rs1333049 (N = 507) polymorphisms on chromosome 9p21, respectively; and the distribution of the genotypes are shown in Table 1. There were significant differences between patients within each polymorphism genotype group for baseline characteristics (Table 1).

**Table 1:** Baseline clinical characteristics according to polymorphisms

	**rs10757274**			***P***	**rs2383206**			***P***	**rs10757278**			***P***	**rs1333049**			***P***
	**N = 498**				**N= 495**				**N= 496**				**N = 507**			
**Variables**	**AA**	**AG**	**GG**		**AA**	**AG**	**GG**		**AA**	**AG**	**GG**		**CC**	**GC**	**GG**	
**Frequency**	112	260	126		84	237	174		115	253	128		139	253	115	
**Age (years)**	60.1±9.3	59.4±9.0	59.3±8.9	0.73	60.5±8.7	59.1±9.3	59.5±9.1	0.50	59.9±9.3	59.5±9.2	59.4±9.3	0.91	59.1±9.2	60.0±9.2	59.4±9.0	0.65
**Men (%)**	65.2	66.9	71.4	0.55	65.5	65.4	71.3	0.42	64.3	66.8	70.3	0.60	70.5	65.6	67.8	0.61
**Smoking (%)**	34.8	28.5	42.9	**0.02**	28.6	31.6	37.4	0.30	32.2	30.4	39.8	0.18	40.3	28.9	33.0	0.07
**Hy (%)**	67.9	55.4	59.5	0.08	73.8	50.2	62.1	**<0.01**	67.0	53.8	60.2	**0.05**	60.4	54.2	65.2	0.12
**DM (%)**	49.1	39.2	29.4	**0.01**	52.4	40.1	32.8	**0.01**	47.8	37.9	32.0	**0.04**	34.5	36.4	49.6	**0.03**
**BMI (kg/m2)**	27.1±4.0	27.2±4.2	27.3±4.0	0.94	27.4±3.9	27.0±4.1	27.4±4.2	0.49	27.3±4.0	26.9±4.1	27.6±4.2	0.26	27.5±4.2	27.0±4.1	27.2±4.2	0.48
**MI (%)**	42.9	42.3	54.0	0.08	41.7	42.6	51.7	0.13	47.8	41.1	53.1	0.07	54.0	40.7	48.7	**0.03**
**TC (mg/dL)**	219.8±48.5	225.5±51.5	222.7±47.0	0.58	220.9±50.5	223.1±51.7	225.8±45.6	0.73	220.0±50.8	226.0±51.8	223.7±44.0	0.56	223.0±43.9	225.7±51.6	219.3±49.9	0.52
**LDLc (mg/dL)**	144.6±45.5	149.9±47.6	144.6±37.0	0.44	144.8±46.8	148.3±47.7	148.3±37.3	0.81	145.7±47.6	149.9±46.7	145.6±35.8	0.58	145.7±36.4	149.6±47.0	145.2±45.6	0.60
**HDLc (mg/dL)**	36.4±10.8	37.1±9.8	38.0±10.1	0.47	35.6±8.7	37.3±10.4	38.2±11.0	0.18	36.3±10.7	37.0±9.4	38.711.7	0.20	38.7±11.5	37.3±10.0	36.0±8.9	0.14
**TG (mg/dL)**	197.7±112.5	194.3±123.8	196.0±110.2	0.96	204.9±116.6	188.3±115.2	195.3±121.5	0.53	194.9±112.7	193.4±115.9	195.1±125.9	0.99	195.1±124.2	191.4±116.0	198.2±112.2	0.87
**PCI (%)**	29.5	33.1	37.3	0.51	27.4	35.0	35.1	0.23	33.9	30.8	39.8	0.30	38.8	32.0	32.2	0.43
**CABG (%)**	32.1	34.2	34.9		36.9	29.5	37.4		29.6	34.8	33.6		35.3	33.6	33.0	
**CT (%)**	38.4	32.7	27.8		35.7	35.4	27.6		36.5	34.4	26.6		25.9	34.4	34.8	
**2-Vessel (%)**	44.6	49.2	31.7	**0.05**	45.2	48.5	36.8	0.06	47.8	47.4	32.8	**0.02**	36.0	46.2	52.2	**0.03**
**3-Vessel (%)**	55.4	50.8	68.3		54.8	51.5	63.2		52.2	52.6	67.2		64.0	53.8	47.8	

*N* = number of samples genotyped.

Data are shown as mean ± standard deviation, unless otherwise indicated.

*Hy* = Hypertension; *DM =* Diabetes mellitus; *MI* = Myocardial infarction; *TC* = Total Cholesterol; *LDLc* = Low density lipoprotein; *HDLc* = High density lipoprotein; *TG* = Triglycerides; *PCI* = Angioplasty; *CABG* = Revascularization; *CT* = Clinical treatment; 2 or 3-vessel = coronary anatomy disease.

The frequency of diabetes was lower in patients carrying GG genotype for rs10757274, rs2383206 and rs10757278 (29.4%, 32.8%, 32.0%) compared to patients carrying AA or AG genotypes (49.1% and 39.2%, p = 0.01; 52.4% and 40.1%, p = 0.01; 47.8% and 37.9%, p = 0.04; respectively). Higher frequency of diabetes was observed in patients carrying GG genotype for rs1333049 (49.6%) compared to patients carrying CC or GC genotypes (34.5% and 36.4%, p = 0.03). Smoking, hypertension and previous myocardial infarction frequencies presented significant difference among rs10757274, rs2383206 and rs1333049 genotypes, respectively (Table 1).

In respect to coronary anatomy, the rs10757274 GG genotype, rs10757278 GG genotype and rs1333049 CC genotype had higher frequencies of triple-vessel disease (68.3%, p = 0.05; 67.2%, p = 0.02; 64.0%, p = 0.03; respectively) (Table 1).

### Cardiovascular end-points

With respect to the end points of death (overall mortality), death from cardiac causes, myocardial infarction, refractory angina requiring either angioplasty or surgery, we observed a higher frequency of overall mortality in patients with the GG genotype for rs2383206 compared to patients with AA and AG genotypes (19.5%, 11.9%, 11.0%, respectively; p = 0.04, Table 2).

**Table 2:** Cardiovascular end-points

	**rs10757274**			***P***	**rs2383206**			***P***	**rs10757278**			***P***	**rs1333049**			***P***
	**N = 498**				**N = 495**				**N = 496**				**N = 507**			
**Events**	**AA**	**AG**	**GG**		**AA**	**AG**	**GG**		**AA**	**AG**	**GG**		**CC**	**GC**	**GG**	
**Refractory angina**	17.9%	18.5%	15.9%	0.82	19.0%	16.0%	17.2%	0.81	19.1%	17.0%	16.4%	0.84	15.8%	17.4%	19.1%	0.79
**PCI**	9.8%	13.8%	9.5%	0.35	9.5%	12.2%	10.3%	0.73	11.3%	11.9%	10.9%	0.96	10.8%	12.6%	11.3%	0.84
**MI**	8.9%	7.7%	7.9%	0.92	10.7%	8.0%	6.3%	0.47	9.6%	7.9%	6.3%	0.63	6.5%	7.5%	9.6%	0.65
**Cardiac death**	8.9%	9.2%	11.1%	0.81	8.3%	7.2%	13.2%	0.11	8.7%	7.5%	13.3%	0.18	11.5%	9.5%	7.0%	0.47
**Total death**	11.6%	13.8%	17.5%	0.42	11.9%	11.0%	19.5%	**0.04**	12.2%	13.0%	17.2%	0.45	15.8%	14.6%	11.3%	0.57
**Total events**	25.9%	29.2%	26.2%	0.73	27.4%	25.3%	27.4%	0.74	26.1%	27.3%	27.3%	0.97	25.9%	28.9%	25.2%	0.71

N = number of samples genotyped.

Refractory angina = requiring either angioplasty or surgery; PCI = angioplasty; MI = myocardial infarction; Cardiac death = death from cardiac causes; Total death = overall mortality; Total events = refractory angina requiring angioplasty or surgery + angioplasty + myocardial infarction + death from cardiac causes + overall mortality.

A Cox proportional hazards model adjusted for age, previous myocardial infarction, diabetes, smoking and type of coronary anatomy (double-vessel or triple-vessel) demonstrated that the rs2383206 was still significantly associated with a 1.75-fold increased risk of overall mortality (p = 0.02) (Table 3). In the same model, age and previous myocardial infarction were associated to a 1.06 and 1.82-fold increased risk of overall mortality, respectively. In addition, age and previous myocardial infarction variables were associated to increased risk of overall mortality in the models including rs10757274, rs10757278 and rs1333049 polymorphisms (Table 3). Gender, hypertension and randomized therapeutic option were also included in the Cox regression models regarding rs2383206, but observed results or increase risk of overall mortality were not changed.

**Table 3:** Cox proportional-hazards model for overall mortality

	***P***	***OR***	**95.0% CI**	
**Age**	**0.00**	1.06	1.03	1.09
**Diabetes mellitus**	0.08	1.51	0.94	2.42
**Smoking**	0.39	1.26	0.74	2.14
**Coronary anatomy**	0.33	1.28	0.77	2.13
**Previous myocardial infarction**	**0.02**	1.76	1.09	2.84
**rs10757274**	0.31	1.31	0.78	2.21
	***P***	***OR***	95.0% CI	
**Age**	**0.00**	1.06	1.03	1.09
**Diabetes mellitus**	0.15	1.41	0.88	2.27
**Smoking**	0.68	1.12	0.65	1.92
**Coronary anatomy**	0.28	1.32	0.79	2.20
**Previous myocardial infarction**	**0.02**	1.82	1.12	2.95
**rs2383206**	**0.02**	1.75	1.08	2.82
	***P***	***OR***	95.0% CI	
**Age**	**0.00**	1.06	1.03	1.09
**Diabetes mellitus**	0.19	1.38	0.85	2.22
**Smoking**	0.77	1.09	0.63	1.89
**Coronary anatomy**	0.17	1.43	0.86	2.40
**Previous myocardial infarction**	**0.02**	1.81	1.11	2.94
**rs10757278**	0.32	1.30	0.78	2.18
	***P***	***OR***	95.0% CI	
**Age**	**0.00**	1.06	1.03	1.09
**Diabetes mellitus**	0.11	1.46	0.91	2.32
**Smoking**	0.29	1.33	0.78	2.24
**Coronary anatomy**	0.15	1.44	0.88	2.37
**Previous myocardial infarction**	**0.03**	1.71	1.06	2.73
**rs1333049**	0.32	1.36	0.74	2.49

Coronary anatomy: double-vessel versus triple-vessel disease.

**Figure 1:**
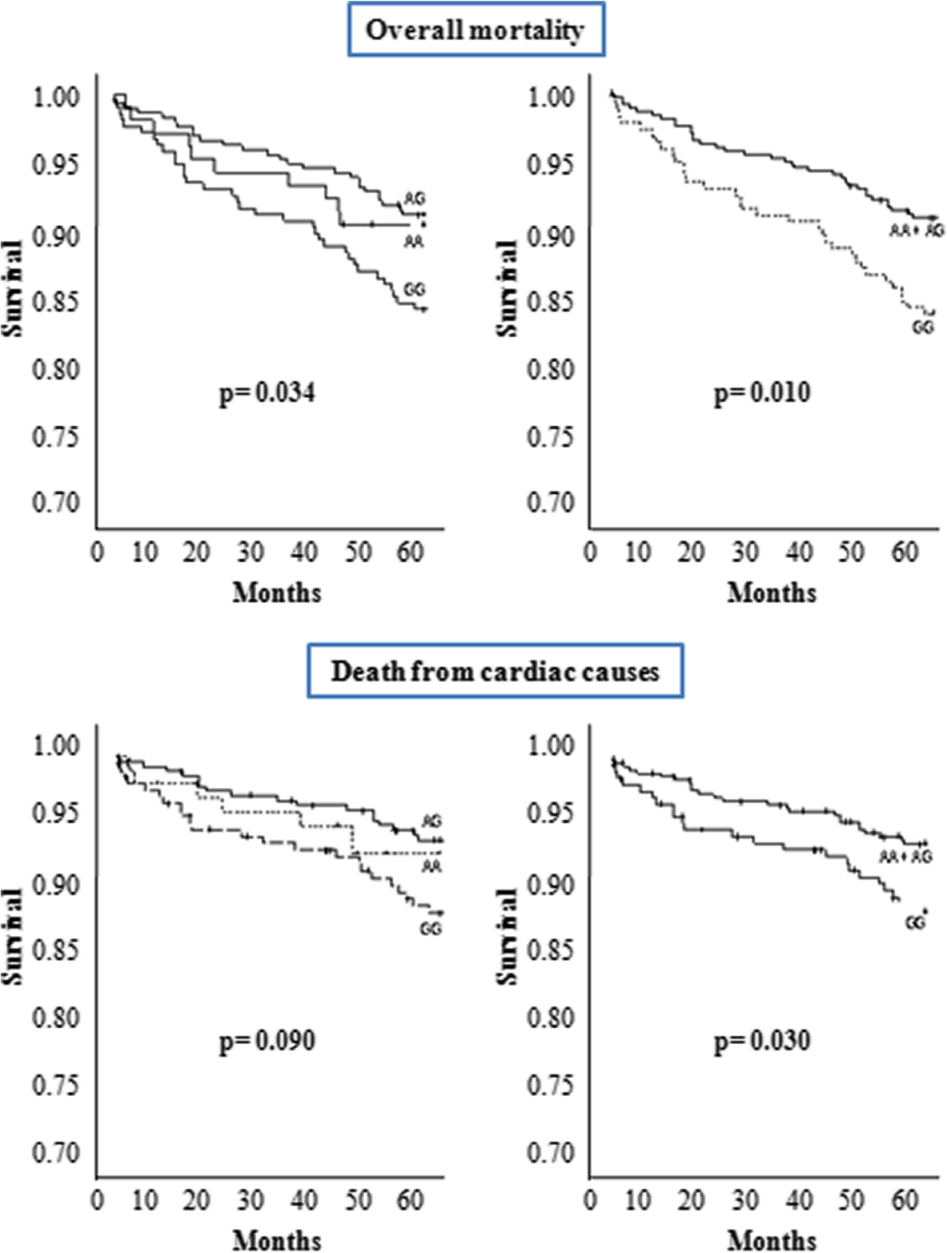
Survival curves for the rs2383206 polymorphism.

In Figure 1, survival curves show significant differences regarding overall mortality for the SNP rs2383206 when we analyze separated (AA, AG, GG; p = 0.034) or grouped genotypes (AA + AG, GG; p = 0.010). We also observed a significant difference for death from cardiac causes among same SNP, but only with grouped genotypes (p = 0.030). In both cases, overall mortality and death from cardiac causes, the GG genotype was associated with increased risk of death.

## Discussion

Significant associations between rs10757274, rs2383206, rs10757278 and rs1333049 polymorphisms and diabetes and coronary artery disease were observed in patients with well-known multi-vessel CAD. Furthermore, our results suggested a significant difference in overall mortality and death from cardiac causes according to the rs2383206 in this patient sample that already presents well established risk factors and multivessel coronary artery disease.

Currently, genome-wide association identified a single region on chromosome 9p21.3 associated with CHD and MI risks [3,4] independently of traditional risk factors including age, gender, obesity, smoking, hypertension and hyperlipidemia [8,9]. Besides, Musunuru et al. have proposed that risk alleles at 9p21.3 locus may have pleiotropic effects on MI, CAD and stroke risks, possibly through their influence on platelet reactivity [23]. Helgadottir et al. [4] identified the rs10757278 that showed significant association with MI risk and that approximately 21% of individuals in the population are homozygous for this variant with estimated risk of suffering MI equal 1.64 times as great as that of non-carriers. McPherson et al. [3] identified 2 SNPs in the same genomic region, rs10757274 and rs2383206, both associated with risk, in 6 independent studies comprising more than 23,000 individuals and with a population-attributable fraction of 10% to 15%. Moreover, the Wellcome Trust Case Control Consortium [24] identified rs1333049 in this region, also showing strong association with CHD risk and a study that combined the Wellcome Trust Case Control Consortium data with data from Germany also confirmed the chromosome 9 region and identified 6 additional novel candidate genes for CHD [12].

In our study, we observed the association of the SNP rs2383206 to CAD within a group of patients with multi-vessel coronary disease that can reveal higher incidence of death depending on its genotype. The reports cited here have provided evidences of increased risk or susceptibility to develop CAD in case–control studies and our findings have showed significant differences of cardiovascular events within a sick population.

Regarding baseline clinical characteristics, our results showed association of all studied SNPs with prevalence of type 2 diabetes mellitus in these patients. In fact, the GG genotypes for the rs10757274, rs2383206 and rs10757278 suggested protection while the GG genotype for the rs1333049 indicated risk. Recent reports have discussed the association of these loci on chr9p21 with type 2 diabetes mellitus suggesting the sharing of pathogenic mechanisms with heart disease [14-16]. Studying the association between the SNP rs2383206 and CAD, Doria et al. observed increased CAD risk in the presence of poor glycemic control in type 2 diabetes [25]. Gori et al. [26] have confirmed that rs2891168 and rs10811661 are independently associated with MI and type 2 diabetes, respectively in an Italian population as reported by the PROCARDIS group [27].

In a recent study, Wang et al. observed a significant association of homozygous CC genotype for the rs1333049 with CAD in non-diabetic Chinese Han population but not in type 2 diabetic patients. Besides, they have demonstrated that CC or GC genotype carriers had a more severe plaque progression than GG genotype carriers [28]. The GRACE genetics study has provided evidence that the association of rs1333049 with recurrent MI or cardiac death was independent of all other risk factors [29]. Another recent report showed that the 9p21 variant rs10116277 is independently associated with all-cause mortality after primary CABG surgery in Whites and significantly improves the predictive value of the logistic EuroSCORE [30]. Here, we find that not only the rs1333049 CC genotype is more associated with serious CAD but also rs10757278 GG genotype; both are related to triple-vessel disease.

Finally, with respect to the end points, there was higher incidence of overall mortality in patients with GG genotype for rs2383206, which remained significantly associated with a 1.75-fold increased risk of overall mortality (CI 95% = 1.08 – 2.82) even after adjustment of a Cox multivariate model. The association of this SNP with death can be observed in the survival curves where not only overall mortality was affected but cardiac death too.

There are some limitations in our study. First, we were not able to perform replication studies in a similar sample because there are very few studies similar to MASSII in the literature. However, our main findings are in accordance to previous studies including a current study published in this journal which confirmed a strong association of the 9p21.3 locus with MI particularly in patients with a positive family history [30]. Second, our sample size only provide 80% power to detect an association with overall mortality with an effect size of 3.4-3.9 for the polymorphisms and correction for multiple testing was not performed. We recognize that such effects are highly unlikely for common genetic variants and reaffirm the need for the development of increased samples of multivessel CAD patients with long-time follow-up period for the identification of more modest effects. In addition, we observed that diabetes and smoking did not have significant effects in the multivariate analysis for overall mortality, as well as gender and hypertension, probably because our sample is composed of more severe patients. Third, it is not possible to completely exclude the interaction between the use of concomitant drugs, other genetic markers, ethnicity and other covariates on our findings [31-34].

## Conclusions

Our data are in accordance to previous evidence that chromosome 9p21 genetic variation may constitute a genetic modulator in the cardiovascular system in different scenarios, including in patients with established CAD, by revealing an association between the rs2383206 and higher incidence of overall mortality and death from cardiac causes in patients with multi-vessel CAD.

## Competing interests

The authors declare that they have no potential conflicts of interest regarding the present publication.

## Authors' contributions

LGP performed the statistical analysis and drafted the manuscript. PCJLS helped to draft and reviewed the manuscript. NEF carried out part of the molecular genetic studies. WAH conceived of the study, and participated in its design. JEK conceived of the study, and participated in its design. ACP conceived of the study, participated in its design and coordination and helped to draft the manuscript. All authors read and approved the final manuscript.

## Pre-publication history

The pre-publication history for this paper can be accessed here:

http://www.biomedcentral.com/1471-2261/12/61/prepub
